# The Stress Barometer: Validation of a Bio–Psycho–Social Brief Screening Instrument of Pandemic Stress Reaction

**DOI:** 10.3389/fpsyg.2022.879535

**Published:** 2022-06-01

**Authors:** Alina Eckhard, Britta Menne, Mareike Salzburger, Martin Poppelreuter, Robert Bering

**Affiliations:** ^1^Centre for Psychotraumatology/Clinic for Psychosomatic Medicine, Alexianer Krefeld GmbH, Krefeld, Germany; ^2^Department of Curative Education and Rehabilitation, Faculty of Human Sciences, University of Cologne, Cologne, Germany; ^3^Specialist Clinic for Psychosomatic, Psychotherapeutic and Internal Medicine, Rehaklinik Glotterbad, Glottertal, Germany; ^4^RehaZentren Baden-Württemberg, Stuttgart, Germany; ^5^Regional Psychiatry Middle-West, Gødstrup, Denmark

**Keywords:** stress barometer, COVID-19, pandemic stress reaction, ICF-orientated, short screening

## Abstract

**Background:**

To capture the psychosocial impact of the SARS-CoV-2 pandemic, a model based on the International Classification of Functioning, Disability, and Health (ICF) was developed during the first lockdown in Germany in April 2020. *FACT-19*, the questionnaire for the assessment of pandemic stress load, measures (1) pre-pandemic stress, (2) pandemic stress, and (3) contextual factors (functioning as facilitators or barriers). Derived from this model, the stress barometer as a brief screening instrument captures these factors. The purpose of this study is a preliminary validation of the instrument.

**Method:**

The stress barometer was applied in conjunction with the Symptom-Checklist SCL-90-S at the beginning of the first lockdown in psychosomatic and psychotraumatological care in two federal states in Germany. The sample consists of *n* = 341 (68.6% female) from 18–73 years of age (*M* = 49.36, *SD* = 11.4).

**Results:**

The structure of the short screening was examined in the overall sample using an exploratory factor analysis [*Ch*i^2^ (78) = 875.720, *KMO* = 0.688]. The results indicate a four-factor-structure that explains 59.5% of the total cumulative variance. The factors of the stress barometer correlate with the Global Severity Index (GSI, measured by SCL-90-S) with moderate to weak effects: pre-pandemic stress (*r*_s_ = 0.431, *p* < 0.001, *n* = 295), pandemic stress (*r*_s_ = 0.310, *p* < 0.001, *n* = 298), distal facilitator (*r*_s_ = −0.155, *p* < 0.001, *n* = 312), and proximal barriers (*r*_s_ = 0.232, *p* < 0.001, *n* = 312).

**Discussion:**

The results indicate the suitability of the stress barometer to complement the measurement of the impact of pandemics with an ICF-oriented approach, taking into consideration pre-pandemic stress as well as interactions with facilitators and barriers. Further analysis will be necessary for a revision of the items of the scale.

## Introduction

As SARS-CoV-2 began to spread, the far-reaching psychosocial impacts of the pandemic quickly became apparent. The protective measures taken to contain the pandemic (e.g., quarantine, contact restrictions) are among others accompanied by social isolation, of which negative consequences for mental health have been proven (Röhr et al., 2020). Moreover, an increase in domestic violence has been recorded since the lockdown began (Kofman and Garfin, 2020). For working parents, school and daycare closure in combination with the shift to working from home represents a double burden of balancing work, childcare, and homeschooling, which at the same time is tightening gender differences (Feng and Savani, 2020). Furthermore, widespread closure of restaurants, retail, and the event industry go along with loss of income leading to economic existential fear (Blustein and Guarino, 2020). All in all, the courses of the disease itself, the impact of protective measures, and fear of infection led to a particular risk suspected among people with mental health disorders (Hossain et al., 2020; Javed et al., 2020; Yao et al., 2020).

Not only existing psychometric instruments have been applied to assess pandemic-related stress reaction since the beginning of the pandemic, but also several newly developed subjective scales have emerged, such as the fear of COVID-19 scale (Bitan et al., 2020), the COVID stress scales (CSS; Taylor et al., 2020), the pandemic stress index (Harkness et al., 2020), and the COVID-19 Pandemic Stress Scale (CPSS; Werner et al., 2021). These scales tend to follow a bio-medical perspective on pandemic-related stress reaction, focusing primarily on one dimension, e.g., fear and anxiety or perceived stress. So far, there has been little empirical attention on bio–psycho–social approaches.

We postulate that due to the dynamic evolution of pandemics, an exclusive consideration of psychological symptom burden or the assignment of diagnosis is not sufficient to assess their psychosocial impact. Instead, a more dimensional, bio–psycho–social approach is necessary to consider pre-existing risk factors as well as resources and barriers alongside the pandemic stress itself (Eckhard et al., 2021a).

### A Bio–Psycho–Social Perspective on Pandemic Stress Reaction

To introduce a bio–psycho–social approach to determine the impact of pandemics, firstly an adequate classification system is needed at the base of a screening instrument. The International Classification of Functioning, Disability, and Health (ICF) was officially endorsed by the World Health Organization (WHO) in 2001 as the international standard for the description and measurement of functioning and disability (World Health Organization, 2002). Since then, the ICF is complementing the bio-medical system of the International Classification of Diseases (ICD).

The base of the ICF is the universally applicable bio–psycho–social model, in which the effects of a health condition (coded according to the ICD) on a person's activity and participation are displayed on interaction with their facilitating or inhibiting contextual factors. Functional health and disability are therefore defined as outcomes of the interactions between health conditions and contextual factors (DIMDI, 2005).

To approach the assessment of pandemic stress reaction from a bio–psycho–social perspective, a model called FACT-19 was developed in orientation to the ICF during the first national lockdown in Germany (Bering et al., 2020a). FACT-19 suggests a triangular model for the measurement of pandemic-related stress reactions (Bering et al., 2020c). It consists of the following three components:

1) Risk factors

Part one of the model is designed to capture pre-pandemic stress burden. Pre-existing risk factors such as traumatic experiences, general medical risk factors, or other stressful life experiences are thought to have a greater influence on the probability of developing long-term health effects after stressful experiences instead of acute psychological symptom burden, and are therefore examined (Bering et al., 2011).

2) Sources of pandemic stress load

The second part of the model aims to identify the individual pandemic-related stress burden. Considering the number of known psychosocial consequences of pandemics, four sources of pandemic stress load are proposed:

a. Lethal threat

Source A, lethal threat summarizes the confrontation of those affected and their relatives with a potentially lethal threat due to an infection with SARS-CoV-2, especially, and not only the elderly and people with previous illnesses are particularly at risk.

b. Economic existential threat

The term economic existential threat (Source B) describes the economic consequences of the pandemic. A widespread shutdown of restaurants, the event industry, and retail go alongside job loss, indebtedness, or insolvency of self-employment, leading to a perceived existential threat.

c. Isolation

In Source C, experiences in connection with isolation due to quarantine and contact restrictions are summarized. For many, the beginning of the pandemic meant balancing working from home with childcare and the assumption of homeschooling due to the closure of schools and daycare. These factors furthermore are accompanied by an increase in conflicts and domestic violence.

d. Fear dynamic

Fear dynamic (Source D) represents concerns and worries related to the pandemic: fear of infection with the virus and its health consequences, as well as worries related to possible social and economic consequences for those affected, their relatives included.

Facilitators and barriers

The third factor of the FACT-19 model represents the interaction with contextual factors, which in accordance with the ICF, can be perceived as moderating or reinforcing the experienced stress burden (e.g., social support and stable job and housing situation vs. lack of social network or debt). In terms of the pandemic, additional contextual factors can be identified: psychoeducation, health behavior education as resources or facilitators vs. visitation bans in hospitals, limited living space, COVID-19 reporting in social media as barriers (Eckhard et al., 2021a).

### The Stress Barometer

Based on this theoretical model, the FACT-19 questionnaire for the assessment of pandemic stress load and the brief screening instrument stress barometer were developed at the beginning of the first national lockdown in Germany. The brief screening instrument, the stress barometer (Bering et al., 2020b), for the immediate and rapid measurement of subjective pandemic stress was implemented in psychotraumatological and psychosomatic care in two clinics in Germany and will be subject to the present study.

Given the psychosocial impact factors of the pandemic, the need for an effective screening instrument with practical applicability became apparent at the beginning of the pandemic. The intention to develop the stress barometer was to create a short, easy-to-perform, subjective questionnaire that can be applied and evaluated in a short amount of time. Furthermore, the aim was to capture not only the acute stress caused by the pandemic and its consequences but to consider the pre-pandemic stress (risk factors that can influence the perceived pandemic impact as well as contextual factors), following hereby a bio–psycho–social approach. The identification of the individual, dominant sources of pandemic stress load serves to efficiently provide the necessary support to those affected.

The present study aims at a preliminary evaluation of the short screening instrument, as well as an investigation of its suitability to measure pandemic stress experiences.

## Methods

### Sample Description

Data were collected from two samples of psychotraumatological and psychosomatic care in Germany. The brief screening instrument was applied to Sample 1 continuously since the first national lockdown in April 2020. In Sample 2, data were collected in the period from July to November 2020. Considering the course of the pandemic, the data collection in Sample 2 started during a period of the pandemic when restrictions were being eased and ended with the beginning of the second national lockdown. Sample 1 consists of patients and rehabilitants of a clinic specializing in psychotraumatological and psychosomatic curative and rehabilitative care in Northrine-Westfalia. Sample 2 was generated in a psychosomatic rehabilitative clinic in Baden-Württemberg.

Participation was solicited based on the following eligibility criteria: a minimum age of 18, written consent to participate in the study, as well as permission to use the data for research purposes. A total of *n* = 377 participants conducted the survey of which *n* = 36 did not complete all questions and therefore were excluded in the present analysis due to incomplete data. Another exclusion criterion was the missing consent to the use of the data for research purposes.

The final overall sample comprised *n* = 341 participants (68.6% female), aged 18–73 years (*M* = 49.36, *SD* = 11.4). Among these, 17.6% were single, 57.77% married, 12.32% were divorced or separated, and 3.23% widowed. Of the participants, 12.32% had not received any vocational training, 63.05% had completed an apprenticeship, and 15.25% had obtained a university degree. At the time of the interview, 61.58% were employed (among these, 34.66% were trained employees and 65.34% were specialists) and 13.20% were unemployed, 1.76% were self-employed, 1.76% homemakers, 2.05% retired, and 7.62% retired due to reduced earning capacity. Of the participants, 2.3% were students at the time of the interview. The sociodemographic characteristics are displayed in Table 1 separately for both samples.

**Table 1:** Sociodemographic variables separated by samples.

	**Sample 1**		**Sample 2**	
	***N* (%)**	***M* (*SD*)**	***N* (%)**	***M* (*SD*)**
Age	130 (38.12 %)	43.7 (13.24)	211 (61.88%)	52.76 (8.49)
Sex				
Female	94 (72.3%)		139 (65.9%)	
Male	36 (27.7%)		72 (34.1%)	
Relationship status				
Single	31 (23.8%)		29 (13.8%)	
Married	64 (49.2%)		133 (63.3%)	
Divorced/separated	14 (10.8%)		28 (13.3%)	
Widowed	5 (3.8%)		6 (2.9%)	
School education^a^				
None	3 (2.3%)		2 (1.0%)	
Hauptschulabschluss	35 (26.9%)		49 (23.3%)	
Mittlere Reife	38 (29.2%)		75 (35.7%)	
(Fach)-Abitur	27 (20.8%)		43 (20.5%)	
(Fach-)Hochschulstudium	4 (3.1%)		30 (14.3%)	
Sonderschulabschluss	4 (3.1%)		0 (0.0%)	
Vocational training				
None	29 (22.3%)		13 (6.2%)	
Completed apprenticeship	72 (55.4%)		143 (68.1%)	
University degree	10 (7.7%)		42 (20.0%)	
Occupation				
Employed (trained activity)	22 (16.9%)		39 (18.6%)	
Employed (Specialist)	28 (21.5%)		87 (41.4%)	
Academic/senior service	4 (3.1%)		27 (12.9%)	
Self-employed	2 (1.5%)		4 (1.9%)	
Homemaker	4 (3.1%)		2 (1.0%)	
Pension	3 (2.3%)		4 (1.9%)	
Disability Pension	15 (11.5%)		11 (5.2%)	
Unemployed	25 (19.2%)		20 (9.5%)	
Student	8 (6.2%)		0 (0.0%)	

a *The schooling system in Germany is divided into primary and secondary education. The primary education consists of the Grundschule (elementary or primary school). The secondary education can be further divided into lower and upper secondary level, including different types of school: The Hauptschulabschluss is the final examination obtained after grade 9 at a Hauptschule. The Realschule on the other hand has a broader range for intermediate pupils. After grade 10 the Mittlere Reife can be obtained as a final examination. The Abitur is the final examination obtained at a Gymnasium after grade 12 or 13, which prepares pupils for higher education. The Sonderschulabschluss is the final examination obtained at a school for children with special educational needs*.

### Materials

The following instruments were applied in the present study.

#### Stress Barometer

The stress barometer is a newly developed brief screening instrument for the measurement of subjective pandemic stress experience. Identifying pre-existing risk factors, the dominant source of pandemic stress and the interaction with contextual factors, the brief screening Stress barometer aims to derive a profile of individual pandemic stress levels, based on which necessary therapeutic interventions and participation-oriented, rehabilitative services can be derived to provide practical support for those affected. Consisting of only ten-staged items, the stress barometer captures pre-pandemic stress (items 1–3, e.g., “How high do you estimate your burden due to physical illnesses before the COVID-19 pandemic?”), sources of pandemic stress (items 4–7, e.g., “How strongly do you feel threatened by economic impacts of the COVID-19 pandemic?”), facilitators and barriers (items 8–10, e.g., “Overall, how supported do you feel by your family?”). The answer scale of the stress barometer is ten-staged. Items 1–3 are rated on a scale from 0 (“not at all burdened“) to 10 (“extremely burdened“). The answer scales from items 4–7 range from 0 (“not at all”) to 10 (“extremely”). Items 8–10 are rated on a scale from 0 (“not at all supported”) to 10 (“extremely supported”). The item contents are displayed in Table 2.

**Table 2:** Factor loading for the items of the stress barometer in the exploratory factor analysis.

**Items**	**I**	**II**	**III**	**IV**	**communality**
**Factors relating to immediate time before COVID-19**
V1: physical illnesses	0.17	−0.154	**0.683**	−0.029	0.52
V2: traumatic experiences	0.123	−0.139	**0.64**	0.088	0.452
V3: stress due to others, not yet mentioned factors	0.083	−0.088	**0.722**	−0.071	0.54
**Factors relating to the impact of COVID-19**
V4: fear of being threatened health wise, economically or socially	**0.849**	−0.075	0.116	0.024	0.74
V5: perceived threat due to contact/travel restriction, isolation or quarantine	**0.726**	−0.033	0.094	0.076	0.543
V6: perceived threat through economic consequences	**0.843**	−0.026	0	−0.01	0.712
V7: perceived lethal threat	**0.642**	0.05	0.239	−0.068	0.476
**Contextual factors**
V8a: facilitator: support from family	−0.13	**0.831**	0.086	−0.086	0.722
V8b: barrier: missing support from family	0.014	**−0.769**	−0.013	0.082	0.598
V9a: facilitator: support from friends and acquaintances	−0.008	**0.737**	−0.095	0.002	0.552
V9b: barrier: missing support from friends and acquaintances	−0.139	**−0.495**	0.333	0.263	0.444
V10a: facilitator: protection due to precautions of the government	−0.195	0.25	0.047	**−0.748**	0.662
V10b: barrier: missing protection due to precautions of government	−0.127	0.003	0.03	**0.87**	0.773
Eigenvalues:	2.868	2.294	1.373	1.201	7.736
Explained variance (%):	22.06	17.64	10.56	9.24	

*basis: n = 308, explained overall variance: 59.5%*.

*Extraction method: principal component analysis, values after Varimax rotation*.

*I = pre-pandemic risk factors, II = pandemic stress burden, III = proximal facilitators/barriers, IV = distal facilitators/barriers*.

*To highlight the assignment of an item to the corresponding factor, the value indicating the factor loading is marked in bold writing*.

#### Symptom-Checklist SCL-90-S

The Symptom-checklist SCL-90-S is a 90-item questionnaire that was applied to assess the subjective physical and psychological symptom burden of the participants over the course of the last 7 days (Franke, 2014). The questionnaire consists of nine subscales (e.g., anxiousness, depressiveness, somatization, and compulsivity) as well as three global scales providing information about the response behavior for all items: the global severity index (GSI) measures basic psychological distress, the positive symptom total (PST) provides information about the number of symptoms for which distress is present, and the positive symptom distress index (PSDI) measures the intensity of the responses. The items are rated on a scale from 0 (“not at all”) to 4 (“very strongly”).

The GSI is considered a particularly good indicator of psychological distress because it summarizes the intensity of perceived distress across all 90 items (Franke, 2014). Therefore, it was selected as a global scale for further data analysis.

### Procedure

Even though the FACT-19 questionnaire and the brief screening instrument stress barometer are based on the same theoretical model, the stress barometer does not consist of shortened items from the FACT-19 questionnaire. Instead, it was developed alongside as a short screening with the purpose of conducting a rapid, subjective overall assessment of the pandemic stress burden. According to the underlying triangular model, the items have been developed to measure a selection of pre-existing risk factors, the four sources of pandemic stress load, and three contextual factors.

The stress barometer and the symptom-checklist SCL-90-S were conducted in a paper–pencil format in both samples. The handling of participants who scored high is based on the individual pandemic stress load. The sources of pandemic stress and participation serve as a starting point for the initiation of necessary therapeutic interventions and participation-oriented, rehabilitative services. For a patient scoring high in Source B, economic existential threat, the instruments of social work and rehabilitation take effect (e.g., direct advice and mediation of state assistance or occupational rehabilitation measures as interventions for patients whose earning capacity is at risk in the long term). Given a dominance in Source C on the other hand, interventions might focus on psychosocial conflict resolution.

Practical support for the identified profile of individual pandemic stress burden was provided in Sample 1 in individual sessions and in Sample 2 in group therapy sessions.

### Data Analysis

#### Exploratory Factor Analysis

To evaluate the structure of the brief screening instrument, an exploratory factor analysis is calculated. The suitability of the selected variables is tested by the inverse correlation matrix, the Kaiser–Mayer–Olkin Measure of sampling adequacy (KMO), and the Bartlett-test. The principal component analysis is chosen as the extraction method. To assess the factor loadings and the assignment of variables to the extracted factors, the rotated component matrix is considered. The resulting factors are then subsequently tested for their internal consistency as a measure of reliability.

#### Spearman's Rho Correlation

To investigate the suitability of the instrument for measuring the stress experience, Spearman's rho is used to evaluate correlations between the components of the stress barometer and the global severity index (GSI). Given the lack of normal distribution of the items (Shapiro–Wilk: *p* < 0.05), the Spearman rho correlation is applied to calculate the linear relationship between the variables as a non-parametric equivalent to the Bravais-Pearson correlation.

#### Regression Analysis

Furthermore, a multiple regression analysis is conducted to check the sample for any influence of sociodemographic parameters (age, gender, relationship status, education) and general symptom burden (GSI), as well as pre-existing risk factors on the pandemic stress load. Firstly, the *F*-test is performed to test whether the regression model is significant and therefore makes an explanatory contribution. Then, the regression coefficients (betas) are checked for significance via *t*-tests, performed for each regression coefficient. The corrected *R*^2^ is used to evaluate how well the estimated model fits the collected data.

#### Descriptive Sample Comparison

Finally, a descriptive comparison of the two samples is carried out. To assess the instrument in different samples and furthermore compare the impact of the pandemic in them, mean differences are compared. Considering that the studied characteristics are not normally distributed in the population of both groups (Shapiro-Wilk: *p* < 0.05), the Wilcoxon-rank sum test is carried out as a non-parametric equivalent to the *t*-Test to check for mean differences in the samples. To assess the significance of the differences in the samples, the effect size (*r*) is calculated.

## Results

### Explorative Factor Analysis: The Structure of the Stress Barometer

The structure of the stress barometer is evaluated by means of an exploratory factor analysis. Considering the preliminary theoretical assumptions, a three-factor structure of the brief screening instrument is expected. The Bartlett-test [Chi^2^ (78) = 875.720, *p* < 0.001] and the Kaiser–Meyer–Olkin value (*KMO* = 0.688) indicate the suitability of the items for the method. Commonly, the *KMO* value is required to be at least 0.60 to proceed with the analysis. According to Kaiser, a value of 0.50 is suggested as the lowest acceptable limit, even though a value above 0.80 is desirable (Kaiser, 1974).

The principal component analysis with varimax rotation suggests the presence of four factors with eigenvalues >1.0. The items of the stress barometer and the corresponding factor loads are displayed in Table 2. On this ground, a four-factor solution is chosen, which explains 59.5% of the cumulative total variance. Accordingly, the measurement accuracy of these is tested via internal consistency as a measurement of reliability: pre-pandemic stress (items 1–3; Cronbach's α = 0.520) and sources of pandemic stress (items 4-7, α = 0.783). For the remaining items, the factor analysis suggests a distinction between proximal (items 8–9) and distal contextual factors (item 10), which due to the bipolarity of the items will be further subdivided into facilitators and barriers. Due to the subdivision of each into two separate items, the item count in Table 2 differs from the original number of items of the screening instrument.

Given that the factors consist of only two items, the measurement accuracy is tested via the Spearman–Brown coefficient. The following values are obtained: proximal facilitators (0.692) and proximal barriers (0.549). Preliminary theoretical assumptions suggest that items 8–10 each correspond to the general component contextual factors. To mark the subdivision indicated by the findings of the factor analysis, in the following they will be called proximal and distal facilitators and barriers.

### Spearman Rho Correlation

To evaluate the suitability of the short screening, its components (pre-pandemic stress, pandemic stress load, and proximal and distal facilitators and barriers) are tested for correlations with the global severity index (GSI) of the symptom checklist SCL-90-S. Due to the bipolarity of the items, facilitators and barriers, that correspond to factors III and IV of the stress barometer, the items are included separately in the correlation analysis.

According to Cohen (1992), significant, moderate effects are confirmed between the GSI and pre-pandemic stress (*rs* = 0.431, *p* < 0.001, *n* = 295), as well as pandemic stress (*rs* = 0.310, *p* < 0.001 *n* = 298). Internal and external facilitators and barriers show significant but weak correlations with the symptom burden. For the components of the brief screening itself weak effects are confirmed between pre-pandemic and pandemic stress (*rs* = 0.299, *p* < 0.001 *n* = 311), pre-pandemic stress and internal barriers (*rs* = 0.120, *p* < 0.05 *n* = 308), as well as for pandemic stress and external facilitator (*rs* = −0.118, *p* < 0.05 *n* = 311). Furthermore, the results indicate strong to moderate effects for contextual factors: internal facilitators and barriers (*rs* = −0.623, *p* < 0.001 *n* = 312), external facilitator and barrier (*rs* = −0.465, *p* < 0.001 *n* = 312). Due to the bipolarity of the items corresponding to the contextual factors, a negative correlation coefficient is reasonable in terms of content. The results of the Spearman rho correlation are displayed in Table 3.

**Table 3:** Spearman rho correlation analysis for components of the stress barometer and the general-severity-index (GSI).

**Items**	***N***	***M* (*SD*)**	**1**	**2**	**3**	**4**	**5**	**6**	**7**
1. pre-pandemic stress	308	5.81 (2.12)							
2. pandemic stress	311	4.82 (2.51)	0.299^**^						
3. internal facilitators	312	2.16 (1.69)	−0.086	−0.082					
4. internal barriers	312	0.732 (1.24)	0.120^*^	0.047	0.623^**^				
5. external facilitator	312	1.56 (1.56)	−0.043	−0.118^*^	0.279^**^	−0.298^**^			
6. external barrier	312	0.485 (1.28)	0.034	−0.059	−0.098	0.251^**^	−0.465^**^		
7. General Severity Index	325	1.33 (0.801)	0.425^**^	0.311^**^	−0.155^**^	0.232^**^	−0.217^**^	0.140^*^	

** *The correlation is significant at the 0.01 level (two-tailed)*.

* *The correlation is significant at the 0.05 level (two-tailed)*.

### Regression Analysis

To evaluate if the variables make an explanatory contribution to the pandemic stress burden, the *F*-test is performed to test whether the regression model is significant. An influence of general symptom burden, pre-existing risk factors, and age on the pandemic stress burden is confirmed [*F*_(6, 266)_ = 9.141, *p* > 0.01, *n* = 272]. The analysis shows that the *t*-tests for the regression coefficient of age (*t* = 2.180, *p* < 0.05), general symptom burden (*t* = 4.539, *p* < 0.01), and risk factors (*t* = 2.596, p <0.05) are significant and therefore have a significant effect on pandemic stress load. The variables sex, school education, and vocational education are not significant and therefore are not further analyzed.

When the general symptom burden increases by one-unit, the pandemic stress burden increases by 0.912 units, holding all other independent variables constant. When risk factors increase by one, pandemic stress increases by 0.193 units. When age increases by one unit (year), pandemic stress increases by 0.29 units, holding all other independent variables constant. The corrected *R*^2^ is = 0.152, meaning that 15.2% of the total variance in pandemic stress burden is explained by general symptom burden, risk factors, and age, which according to Cohen (1992) corresponds to a medium effect.

### Descriptive Sample Comparison

The results of the descriptive sample comparison are displayed in Table 4. Sample 1 shows a higher amount of pre-pandemic traumatic experiences (Mdn = 8) than sample 2 (Mdn = 6): *Z* = −4.74 and *p* < 0.001. The effect size according to Cohen (1992) is *r* = 0.34 and corresponds to a moderate effect. Small effects become apparent in the overall pre-pandemic stress (Sample 1: Mdn = 6.34, Sample 2: Mdn = 5.67; *Z* = −2.24, *p* < 0.05, *r* = 0.13), as well as in terms of pandemic stress due to isolation experiences (Sample 1: Mdn = 6, Sample 2: Mdn = 5; *Z* = −2.08, *p* < 0.05, *r* = 0.12).

**Table 4:** Descriptive sample comparison for the components of the stress barometer.

**Items**	***N***	***Mdn***	***Z***	***p***	***r***
*Overall pre-pandemic stress*			**−2.24**	**<0.05**	**0.13**
Sample 1	109	6.34			
Sample 2	199	5.67			
physical illnesses			−1.35	>0.05	
Sample 1	109	6			
Sample 2	199	7			
Traumatic experiences			**−4.74**	**<0.001**	**0.34**
Sample 1	109	8			
Sample 2	200	6			
Stress due to others, not yet mentioned factors			−0.06	>0.05	
Sample 1	106	6			
Sample 2	193	6			
*Overall pandemic stress*			−0.331	>0.05	
Sample 1	109	5			
Sample 2	202	5			
fear of being threatened health wise, economically, or socially			−0.72	>0.05	
Sample 1	109	5			
Sample 2	202	6	
Perceived threat due to contact/travel restriction, isolation or quarantine			**−2.08**	**<0.05**	**0.012**
Sample 1	110	6			
Sample 2	202	5			
perceived threat through economic consequences			−0.93	>0.05	
Sample 1	110	5			
Sample 2	202	5			
Perceived lethal threat			−0.82	>0.05	
Sample 1	109	5			
Sample 2	202	5			
*Contextual factors*
Proximal facilitators			−0.945	>0.05	
Sample 1	110	2			
Sample 2	202	2			
Proximal barriers			**−5.53**	**<0.001**	**0.31**
Sample 1	110	0.5			
Sample 2	202	<0.01			
Distal facilitator			**−3.43**	**<0.01**	**0.19**
Sample 1	110	<0.01			
Sample 2	202	2			
Distal barrier			**−3.76**	**<0.001**	**0.21**
Sample 1	110	2			
Sample 2	202	<0.01			

*Bold values indicate significant results*.

Furthermore, differences in the samples are observed for contextual factors. Sample 1 (Mdn = 0.5) shows a higher number of internal barriers than Sample 2 (Mdn <0.01), indicating moderate effects with *Z* = −5.53, *p* < 0.001 and *r* = 0.31). In contrast, external facilitators are higher in Sample 2 (Mdn = 2) than in Sample 1 (Mdn = 0.00), showing small effects (*Z* = −3.43, *p* < 0.01, *r* = 0.19).

## Discussion

The purpose of this study was to provide an initial review of the application-oriented brief screening for pandemic stress exposure. The findings suggest that the underlying construct of the stress barometer postulating the components pre-pandemic stress, pandemic stress, and contextual factors can be partly confirmed. Instead of assigning item 10 to contextual factors as presumed, the results of the explorative factor analysis indicate a further subdivision of this component into proximal (social and family support) and distal contextual factors (e.g., precautions of the government). The hereby emerging four-factor solution explains 59.5% of the overall variance. Considering preliminary theoretical assumptions as in the bio–psycho–social model, we postulate that proximal and distal facilitators and barriers correspond to the more general term of contextual factors.

The Spearman rho correlation between the components of the brief screening instrument and the *global severity index* serves as a preliminary evaluation of the suitability of the stress barometer to measure subjective stress burden. From the factors of the instrument, the highest correlation is observed between pre-pandemic stress and the GSI. The factor pre-pandemic stress evaluates the existing stress burden in the immediate time before the outbreak of the pandemic, which functions as a risk factor. The higher the risk factors, the higher seems to be the general symptom burden. Preliminary theoretical assumptions postulate the interaction of contextual factors with stress burden. Functioning as facilitators or barriers, contextual factors are thought to have a moderating or aggravating influence on the impact of the individual. Considering the findings of this analysis, the strongest correlation becomes apparent between facilitators and barriers. The deriving question is, why do contextual factors influence relatively little the symptom burden? Earlier investigations have shown that functional levels serve as a more adequate parameter to indicate long-term effects than the actual symptom burden (Bering et al., 2011). Further analysis will be necessary to examine functional levels, e.g., the global assessment of functioning (GAF) (Aas, 2011).

The multiple regression analysis confirmed that 15.2% of the total variance in pandemic stress burden is explained by general symptom burden, risk factors, and age.

To investigate the consistency of the short screening, a sample comparison is conducted. The analysis shows small to no differences in the factors of the stress barometer between the samples, indicating similar findings of the short screening in both samples.

## Limitations

The following limitations of the present study must be considered in the interpretation of the results. First, the sample sizes are relatively small with unequal group sizes. Second, the KMO measure indicates that the data are only moderately suitable for the application of the factor analysis. Similarly, the values of internal consistency for the factors of pre-pandemic stress and proximal barriers are low. Therefore, further analysis for a revision of the items of the scale will be necessary.

The descriptive sample comparison indicates similar findings of pandemic stress burden in both samples. The differences noted between the samples can be partly attributed to clinical differences, e.g., the greater number of pre-existing traumatic experiences can be explained due to the psychotraumatologic specialization of the clinic from Sample 1. However, the overall comparability of the samples is limited due to various reasons. On the one hand, the samples differ due to the specialization of the clinics. Sample 1 consists of patients in curative care and rehabilitants in the rehabilitative care, while Clinic 2 focusses on rehabilitative care. More importantly and regarding the dynamic development of the pandemic, data were collected at different times during its course. The survey in Sample 1 was conducted at the beginning of the first lockdown. Sample 2, however, was surveyed at a time between two waves of the pandemic, where at the beginning of the survey the restrictions were eased. The survey ended with the beginning of the second national lockdown. An influence in the form of experiences with the pandemic and knowledge gained about protective measures or the expected course of the pandemic on the pandemic stress burden collected cannot be excluded.

Furthermore, the participants reported pre-pandemic risk factors retrospectively.

## Conclusion

Further studies will be necessary to approach the limitations in the present study as well as a revision of the items, especially in terms of the components pre-pandemic stress and contextual factors. Yet, the advantage of the short screening is apparent: The stress barometer provides a solution to the previously existing problem of the lack of a specific instrument to assess the psychosocial impact of the COVID-19 pandemic. Contrary to other investigations, instead of applying or revising an existing psychometric instrument, a new instrument was developed. In comparison to other newly developed subjective scales focusing on a bio-medical approach, the stress barometers specifically capture the individual pandemic impact based on the bio–psycho–social model of the ICF. Hereby, the brief screening instrument meets the German obligatory requirement set by the Federal Participation Act and the Ninth Social Code to determine the need for participation based on the ICF (Art. 1, 26 BTHG in accordance with §118 SGB IX).

Instead of offering a large test battery, the brief screening stress barometer can be applied and evaluated by different occupational groups in the health care system within a few minutes and without greater effort. Taking into consideration pre-pandemic stress as well as interactions with facilitators and barriers, the short screening gives first indications of a possible risk profile. Its advantage, therefore, lies in the immediate consideration of the therapeutic curse. Necessary therapeutic interventions (e.g., psychosocial support, conflict counseling, and mediation of government aid) can be derived based on the dominant source of pandemic stress and can be initiated in a timely manner to prevent deterioration (Eckhard et al., 2021b).

So far, the brief screening instrument has been mainly applied to people with pre-existing mental health conditions. Nonetheless, the scale can be suitable for the early detection of people in the general population who may be at risk. A pilot study was conducted in the social environment of students at a German university. At the present, results are not available yet, and further analysis of participants from the general population will be necessary.

All in all, the results indicate the suitability of a bio–psycho–social approach on the measurement of pandemic stress burden. The advantage of this more-dimensional perspective lies in the assessment of pre-existing risk factors, as well as resources and inhibiting factors alongside the pandemic impact itself. By identifying the dominant sources of pandemic stress, immediate therapeutic intervention can be deduced and applied. With further analysis, the stress barometer as a brief screening instrument with an ICF-oriented approach can complement the measurement of the impact of the SARS-CoV-2 pandemic.

## Data Availability Statement

The datasets presented in this article are not readily available because of ethical reasons (patient confidentiality/participant privacy). Requests to access the datasets should be directed to AE, aeckhar2@uni-koeln.de.

## Ethics Statement

Ethical review and approval was not required for the study on human participants in accordance with the local legislation and institutional requirements. The patients/participants provided their written informed consent to participate in this study.

## Author Contributions

RB developed the theoretical model and the questionnaire. AE contributed to the further development of the screening instrument, organized the database of Sample 1, and organized the database of the overall sample and performed the statistical analysis. RB and AE contributed to the conception and design of the study and coordinated the data collection in Sample 1. BM, MS, and MP contributed to the design of the study in Sample 2, coordinated the data collection, and organized the database of Sample 2. All authors contributed to manuscript revision, read, and approved the submitted version.

## Funding

We acknowledge support for open access publishing from the DFG (German Research Foundation, 491454339).

## Conflict of Interest

AE was employed by Alexianer Krefeld GmbH, Germany. The remaining authors declare that the research was conducted in the absence of any commercial or financial relationships that could be construed as a potential conflict of interest.

## Publisher's Note

All claims expressed in this article are solely those of the authors and do not necessarily represent those of their affiliated organizations, or those of the publisher, the editors and the reviewers. Any product that may be evaluated in this article, or claim that may be made by its manufacturer, is not guaranteed or endorsed by the publisher.
